# Biomolecules with Antioxidant Capacity from the Seeds and Sprouts of 20 Varieties of *Chenopodium quinoa* Willd. (Quinoa)

**DOI:** 10.3390/plants10112417

**Published:** 2021-11-09

**Authors:** Edwin Carlos Enciso-Roca, Enrique Javier Aguilar-Felices, Johnny Aldo Tinco-Jayo, Jorge Luis Arroyo-Acevedo, Oscar Herrera-Calderon

**Affiliations:** 1 Department of Human Medicine, Faculty of Health Sciences, Universidad Nacional de San Cristobal de Huamanga, Portal Independencia 57, Ayacucho 05003, Peru; edwin.enciso@unsch.edu.pe (E.C.E.-R.); enrique.aguilar@unsch.edu.pe (E.J.A.-F.); johnny.tinco@unsch.edu.pe (J.A.T.-J.); 2 Department of Dynamic Sciences, Faculty of Medicine, Universidad Nacional Mayor de San Marcos, Av. Miguel Grau 755, Lima 15001, Peru; jarroyoa@unmsm.edu.pe; 3 Department of Pharmacology, Bromatology and Toxicology, Faculty of Pharmacy and Biochemistry, Universidad Nacional Mayor de San Marcos, Jr. Puno 1002, Lima 15001, Peru

**Keywords:** Amaranthaceae, free radical, superfoods, phytochemical analysis, flavonoids, phenols, amino acids

## Abstract

Quinoa has acquired a great interest due to its high content of nutrients and biomolecules that have nutritional and medicinal properties. The aim of this study was to compare the total phenolic content (TPC), total flavonoids (TF), and the antioxidant capacity of 20 varieties of seeds and sprouts of quinoa extract. Quinoa seeds were germinated for 72 h and dried in an oven at 45 °C. The extracts were obtained by dynamic extraction using methanol. Phytochemical analysis with liquid chromatography coupled with mass spectrometry (LC-ESI-MS/MS), TPC, TF, and the antioxidant capacity was carried out and compared between both extracts. The TPC was determined with Folin-Ciocalteu reagent, TF with AlCl_3_, and the antioxidant capacity was determined according to the DPPH and ABTS assays. Sprout extracts showed high values of TPC (31.28 ± 0.42 mg GAE/g; Pasankalla variety), TF (14.31 ± 0.50 mg EQ/g; black Coito variety), and antioxidant capacity (IC_50 (DPPH)_: 12.69 ± 0.29 µg/mL and IC_50 (ABTS):_ 3.51 ± 0.04 µg/mL; Pasankalla). The extracts of the Pasankalla variety revealed 93 and 90 phytochemical constituents in the seeds and sprouts, respectively, such as amino acids, phenolic acids, flavonoids, fatty acids, and triterpene saponins, among others. Quinoa sprouts showed a high content of TPC and TF, and high antioxidant capacity compared with seed extracts, especially the Pasankalla variety.

## 1. Introduction

Quinoa (*Chenopodium quinoa* Willd.) is a pseudocereal belonging to the Amaranthaceae family that is native to the Andean region in South America [1]. Peru is the leading quinoa-exporting country, exporting quinoa with a value of $98.5 million dollars, followed by Bolivia, the Netherlands, the United States, Spain, Germany, Canada, France, Ecuador, and Belgium [2]. Quinoa seeds are known to have a high protein content ranging from 11% to 19%. The seeds are a source of amino acids (isoleucine, leucine, lysine, methionine, phenylalanine, threonine, tryptophan, valine, histidine, cysteine, tyrosine, glycine, arginine, proline, serine, glutamine, alanine, and aspartic acid), carbohydrates (49% to 68% dry weight), fat (2% to 9.5%), vitamins (thiamine, riboflavin, folic acid, and niacin), and minerals such as iron, zinc, magnesium, and copper (2.4% to 4.8%) [3]. Additionally, some phytochemical constituents such as saponins, phenolic compounds (ferulic, sinapinic and gallic acids, kaempferol, isorhamnetin, and rutin) [4], and peptides with therapeutic activity have been determined, making this crop very attractive for a wide range of food products [5]. Quinoa has been traditionally used in tortillas, pasta, flour, cookies, bread, and soups, among others, and is considered to be a gluten-free superfood and a source of fiber dietary [6]. Thus, quinoa is considered to be an acceptable food worldwide and is highly recommended for vegetarians.

On the other hand, sprouts are obtained by germinating the seeds and provide multiple nutritional and therapeutic benefits to those who consume them in different ways, due to the increase in the availability of nutrients such as fatty acids and carbohydrates, as well as polyphenols and flavonoids, during the germination process, which improves their antioxidant capacity [7]. These changes are due to a multitude of biochemical processes, which generate alterations in the composition of primary and secondary metabolites, producing an intrinsic change in the phenolic compounds and antioxidant activity [8]. Sprouts can improve the nutritional quality of a grain by eliminating or inactivating some antinutritional factors and increasing the digestibility of proteins and starches [9]. During germination, the original composition of the seed changes: the nitrogen-containing proteins move towards smaller protein fractions, oligopeptides, and free amino acids (some increase; others decrease or are not altered). Consequently, the changes increase the biological protein value of the sprouts, and digestibility is higher than in seeds [10]. 

Studies have reported that quinoa sprouts have high levels of amino acids, peptides, vitamins, and minerals but also include antinutritional components such as tannin, lectin, trypsin inhibitor, and galactoside, although at lower values than in non-germinated seeds [11]. The main enzyme involved in the early phase during the sprouting of quinoa seeds seems to be α-amylase, which leads to the generation of new compounds [12]. Some biological studies in quinoa sprouts have reported hepatoprotective, antioxidant [13], and anti-α-amylase effects in vitro [14], and hypoglycemic effects in diabetic rats [15]. Currently, there are no studies on the antioxidant activity of a wide variety of quinoa sprouts grown in Peru. Thus, as the germination process is a strategy for obtaining sprouts and improving the antioxidant capacity, total phenols, and flavonoids, thereby increasing its nutraceutical value, the main aim of this study was to compare the total phenolic content, flavonoids, and antioxidant capacity of the seeds and sprouts of 20 varieties of quinoa and analyze the phytochemical constituents of varieties with major antioxidant capacity using liquid chromatography-mass spectrometry (LC-ESI-MS/MS). To carry out this study, four phases were developed:

(a) Germinating 20 varieties of quinoa seeds under laboratory conditions and extracting their phytochemical constituents by maceration with methanol.
(b) Determining the total phenolic content (TPC) and total flavonoids (TF) of the seeds and sprouts of quinoa.
(c) Evaluating the antioxidant capacity of the seeds and sprouts of quinoa using the 2,2-diphenyl-1-picrylhydrazyl (DPPH) and 2,2′-azinobis-(3-ethylbenzothiazoline)-6-sulfonic acid (ABTS) methods.
(d) Analyzing the phytochemical constituents of the seeds and sprouts of the quinoa variety with the best results obtained regarding the antioxidant capacity with liquid chromatography-mass spectrometry (LC-ESI-MS/MS).

## 2. Results

### 2.1. Germination Process

**Figure 1:**
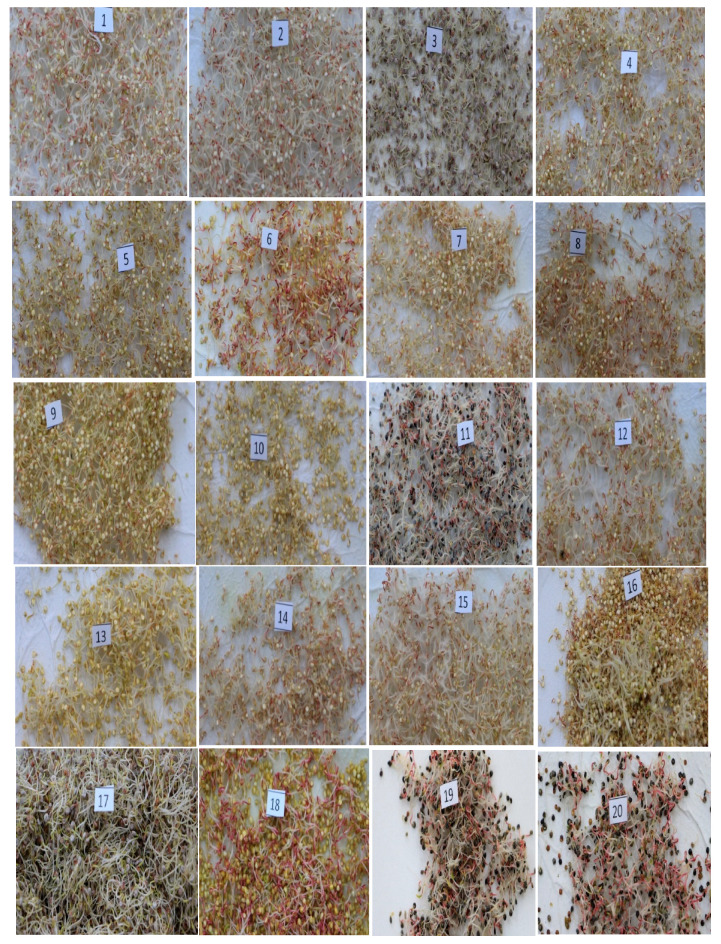
Twenty varieties of quinoa sprouts. (**1**), White Junín Ayacucho; (**2**), T-256; (**3**), Pasankalla; (**4**), Suano Puno; (**5**), T-38; (**6**), Yellow Sacaca; (**7**), T-45; (**8**), Santa Ana; (**9**), T-61 Pomata; (**10**), CQA-048; (**11**), Black Collana; (**12**), T-72 Huancayo; (**13**), CQA-043; (**14**), Salcedo; (**15**), Ayacucho Compuesto; (**16**), White Choclito; (**17**), Red; (**18**), Yellow Maranganí; (**19**), Black Coito; (**20**), Black.

Sprouts were obtained in a time of 72 h, and measured between 1.7 and 2.3 cm in length for all varieties. However, the red variety achieved the greatest length among all varieties (2.1–2.3 cm). The other varieties had lengths as follows: White Junín Ayacucho, 1.7–1.9 cm; T-256, 1.8–1.9 cm; Pasankalla, 1.7–1.8 cm; Suano Puno, 1.7–1.9 cm; T-38, 1.8–2.0 cm; Yellow Sacaca, 1.9–2.0 cm; T-45, 1.7–1.9 cm; Santa Ana, 1.7–1.8 cm; T-61 Pomata, 1.8–1.9 cm; CQA-048, 1.8–2.0 cm; Black Collana, 1.7–1.9 cm; T-72 Huancayo, 1.8–1.9 cm; CQA-043, 1.8–1.9 cm; Salcedo, 1.8–2.0 cm; Ayacucho Compuesto, 1.7–1.9 cm; White Choclito, 1.7–1.9 cm; Yellow Maranganí, 1.9–2.1 cm; Black Coito, 1.7–1.9 cm; and Black, 1.8–2.0 cm. Figure 1 shows the 20 varieties of quinoa germinated under standard laboratory conditions of temperature, humidity, and time.

### 2.2. Total Phenolic Content

The TPC of sprouts was found to range from 19.15 ± 1.54 to 31.28 ± 0.42 mg GAE/g of methanolic extract, being highest in the Pasankalla variety, CQA-048, Black Collana, and Black Coito. On the other hand, in quinoa seed extracts, the variation was from 11.72 ± 0.32 to 28.32 ± 0.49, being greater in the Pasankalla, Black Collana, and Black Coito varieties (Table 1). There was a significant difference between sprout and seed extracts for TPC, (paired sample t-test; *p* < 0.05), with TPC being higher in sprout extracts than in seed extracts, with an average of 24.57 ± 3.49 mg GAE/g in sprout extracts and 20.12 ± 4.37 mg GAE/g in seed extracts.

### 2.3. Total Flavonoids

In sprouts, the flavonoid content varied from 7.44 ± 0.50 to 14.31 ± 0.5 mg EQ/g of extract, being highest in the Black Coito, Yellow Maranganí, Pasankalla, and Black Collana varieties. In seed extract, the variation was from 6.23 ± 0.26 to 11.52 ± 0.92 EQ/g. The results showed a significant increase in the total flavonoids in sprouts compared with seed extracts, but in some varieties, the increase was not significant, such as in T-256, Suano Puno, T-38, CQA-043, and Ayacucho Compuesto (Table 1).

### 2.4. The Antioxidant Capacity Equivalent to Trolox 

Table 2 shows the antioxidant capacity equivalent to Trolox (TEAC) of sprout and seed extracts, with the variation ranging, respectively, from 25.90 to 37.65 and from 25.03 to 29.60 µmol ET/mg of extract for the radical DPPH and from 57.05 to 90.84 and from 45.80 to 67.04 µmol ET/mg of extract for the radical ABTS. The sprouts with the highest antioxidant capacity for the radical DPPH were Pasankalla, White Junín Ayacucho, Yellow Sacaca, Black Collana, and Red; while for the radical ABTS, those with the highest antioxidant capacity were Black Collana, Black, Pasankalla, Suano Puno, Yellow Maranganí, Red, and Black Coito. Furthermore, it was found that in most of the varieties, significant differences appeared (Student’s *t*-test; *p* < 0.05), with antioxidant capacity being greater in sprouts than in seed extracts. 

### 2.5. The Half Inhibitory Concentration (IC_50_) of the Methanolic Extracts of Sprouts and Seeds of Quinoa

The half inhibitory concentration (IC_50_) (Table 3) represents the reduction to 50% of the initial absorbance of the DPPH and ABTS radicals, with the average variation for all varieties ranging from 12.69 to 18.45 mg/mL in sprout extracts and from 16.15 to 19.09 mg/mL in seed extracts using the DPPH assay. In the ABTS assay, the results ranged from 3.05 to 4.71 and from 4.13 to 6.04 mg/mL in sprout and seed extracts, respectively. There was a significant difference (*p* < 0.05) in the IC_50_ of the radicals DPPH and ABTS between the sprout and seed extracts, being lower in sprouts than in seed extracts.

Table 4 shows positive correlations between antioxidant capacity and both TPC and total flavonoids, and a negative correlation with IC_50_, with a significant difference in both cases (*p* < 0.01). This correlation indicates that while the concentration of TPC and TF increased in sprout extracts, their antioxidant capacity also increased and, inversely, as TPC and TF became higher, the IC_50_ reduced.

### 2.6. Phytochemical Analysis of Methanolic Extracts of Sprouts and Seeds of C. quinoa (Pasankalla Variety)

**Figure 2:**
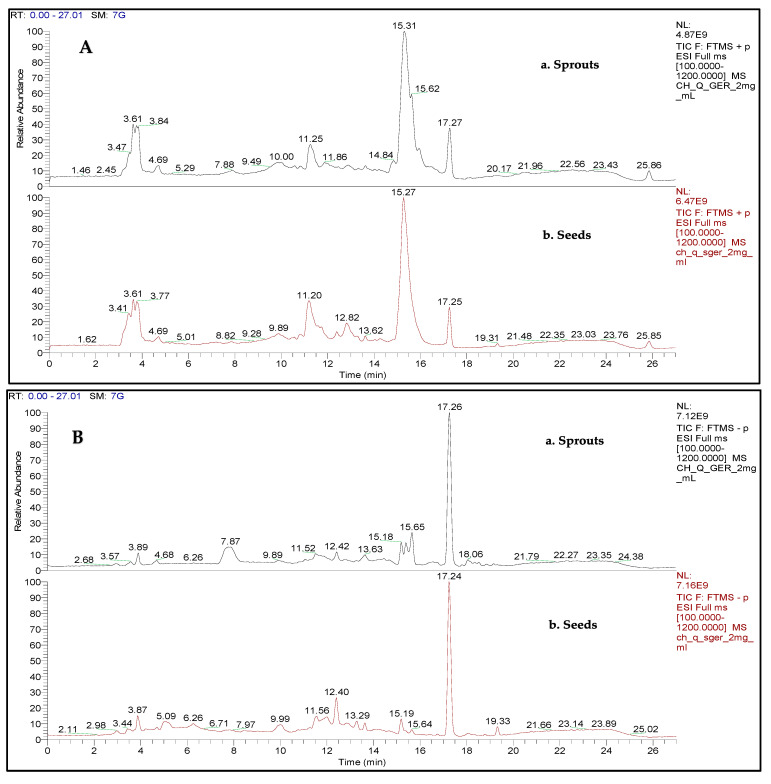
Chromatographic profile (LC-MS) of C. quinoa extracts (Pasankalla variety): (**A**): ESI (+) ionization mode; (**B**): ESI (−) ionization mode.

Phytochemical analysis was carried out by LC-ESI-MS/MS for the Pasankalla variety due to its high TPC and TF values and antioxidant capacity, as shown in Table 1, Table 2 and Table 3. Our results indicated that the sprout extract had 90 phytochemical constituents, of which 45 were observed in ESI (−), 33 in ESI (+), and 12 in both modes. In the seed extract, 93 compounds were determined, of which 58 metabolites were observed in ESI (−), 28 in ESI (+), and 7 in both modes, as presented in Table 5. Figure 2 shows the ESI-positive and -negative chromatographic profiles for both sprouts and seeds of the Pasankalla variety.

The retention times (Rt), adductions, experimental, and theoretical m/z values, ppm error, MS/MS spectrum (m/z: absolute intensity), SMILES (simplified molecular input line entry system) string, InChIKey (IUPAC international chemical identifier), and tentative compounds are available in the Supplementary Table S1 and Supplementary Table S2. 

The phytochemical constituents determined in the extracts of Pasankalla sprouts (Table 6) were classified as (i) primary metabolites, such as amino acids and derivatives (n = 23), organic acids (n = 14), monosaccharide sugar acids and sugar alcohols (n = 8), disaccharides and oligosaccharides (n = 7), lipids (n = 8), and nucleobases/nucleosides (n = 5); and (ii) secondary metabolites, such as phenolic acids (n = 2), triterpenoids (n = 4), O-glycosyl compounds (n = 4), phenolic glycosides (n = 2), flavonoid-O-glycosides (n = 2), alkaloids and derivatives (n = 1), triterpene saponins (n = 4), coumarins (n = 1), and other compounds (n = 13). 

In the seeds (Table 7), the phytochemical constituents were classified as: (i) primary metabolites, such as amino acids and derivatives (n = 11), organic acids (n = 16), monosaccharides sugar acids and sugar alcohols (n = 8), disaccharides and oligosaccharides (n = 7), lipids (n = 14), and nucleobases/nucleosides (n = 5); and (ii) secondary metabolites, such as triterpenoids (n = 3), catechols (n = 3), phenolic glycosides (n = 2), flavonoids (n = 5), alkaloids and derivatives (n = 1), triterpene saponins (n = 1), and other compounds (n = 17). 

## 3. Discussion

Polyphenolic compounds are secondary metabolites present in plants, which are divided into flavonoids and non-flavonoids, the first being responsible for the antioxidant capacity, exerting this through various mechanisms such as transition metal chelators, free radical scavengers, and enzyme inhibitors [16]. The antioxidant properties of secondary metabolites are related to vasodilatory, lipid-lowering, antiaging, and anti-inflammatory, modulating apoptosis processes in the vascular endothelium, but these molecules could also be influenced by factors such as the number and position of the phenolic hydroxyl groups, steric effects, and molecular properties [17]. In our results, the content of total phenols and flavonoids found in quinoa sprouts presented differences in each variety analyzed, being influenced by the type of seed, the cultivation site, maturity, storage, and germination conditions, as the flavonoids play an important role in pigmentation [18]. It is known that the phenolic compounds present in plants are formed during their development and under stress conditions; these include simple phenols, phenolic acids, coumarins, flavonoids, stilbenes, hydrolysable and condensed tannins, lignans, and lignins [19]. Additionally, these polyphenols could be altered during the germination process, increasing their content and the antioxidant capacity [20].

In our study, the variation in TPC and TF differed from the studies of Valencia et al., in which the TPC varied from 0.783 to 3437 mg GAE/g in quinoa seeds [21], and that of Carciochi et al. [22], with values of TPC of 39.3 ± 0.9 mg GAE/100 g and TF of 11.06 mg of quercetin/100 g in sprouts. These were higher in our study due to the type of solvent used in the maceration process. In the same way, when the antioxidant activity of the content of polyphenols and flavonoids was evaluated in the red and yellow varieties of quinoa, there was a significant increase after 9 days of germination. In a similar study, the antioxidant capacity in germinated seeds was greater compared with seeds of *C. quinoa*, increasing up to twofold, similar to the increase in phenolic compounds and antioxidant capacity observed after 72 h of germination [13]. In our study, a wide range of values were observed for phenolic compounds and flavonoids, as well as for the antioxidant activity in each variety of quinoa studied, which can be explained by the characteristics of each seed, variation in the availability of nutrients, and activation of the antioxidant machinery during germination. 

Several studies have shown nutritional improvements in quinoa sprouts, such as in crude quinoa flour (CQF) and germinated quinoa flour (GQF), where the CQF/GQF ratio increased the nutritional quality of pasta. Chemical analysis indicated an increase in the proportion of proteins by 37% and a decrease in phytic acid by 77%, which means that the germination process is an effective method to minimize phytic acid content in seeds. Pasta with a high CQF/GQF ratio had an increased content of Ca, K, Fe, Mn, Mg, P, and Zn, and thus using GQF is recommended in the production of bread, cakes, and cookies to take advantage of their nutritional properties, which provide a high content of proteins, minerals, TPC, and amino acids, and a low amount of phytic acid [23]. During germination, quinoa seeds undergo relevant physical and chemical changes; the maximum intensity of macromolecular modification occurs at 48 h. The germinated material contains micronutrients with improved bioavailability. This has a great impact on quinoa, as it improves the technological properties of quinoa, as well as some of its nutritional characteristics, enhancing the use of quinoa sprout flour as an ingredient in food formulation [12]. The germination process of quinoa seeds is an effective technique to enhance the content of total phenols and total flavonoids and to improve the antioxidant capacity, as was demonstrated in quinoa (*C. quinoa*) and kiwicha (*Amaranthus caudatus*) [24], where the sprouts had enhanced content of coumaric acid and kaempferol tri-glycoside in quinoa and caffeoylquinic acid in kiwicha. Additionally, a significant increase was observed in the phenolic content and the antioxidant capacity through malting quinoa sprouts [25] and *Amaranthus caudatus* sprouts [26]. 

## 4. Materials and Methods

### 4.1. Collection of Quinoa Seeds

Fifteen certified varieties were provided by the Agrarian Research Institute (INIA, Ayacucho, Peru) and five varieties were collected between November and December 2019 in the districts of Huamanguilla and Acocro of the province of Huamanga. These are registered with the following names: White Junín Ayacucho, T-256, Pasankalla, Suano Puno, T-38, Yellow Sacaca, T-45, Santa Ana, T-61 Pomata, CQA-048, Black Collana, T-72 Huancayo, CQA-043, Salcedo, Ayacucho Compuesto, White Choclito, Red, Yellow Maranganí, Black Coito, and Black. 

### 4.2. Germination Process

The seeds were washed with hypochlorite 0.02% (*w*/*v*) for 20 min, rinsed several times with distilled water, and placed on absorbent paper moistened with distilled water in Technopor containers covered with paper towels and incubated at room temperature (between 18 and 22 °C) for 72 h until good sprouts had been obtained. The sprouts were harvested, dried at 45 °C for 48 h, then crushed and stored under refrigeration [27].

### 4.3. Preparation of the Methanolic Extract 

Ten grams of each sample of sprouts and seeds was subjected to dynamic extraction with 100 mL of methanol (1:10), using a magnetic stirrer for 4 h at room temperature, then filtered with Whatman No. 1 paper and concentrated on a rotary evaporator until dry. Each extract was refrigerated until further use at 4 °C.

### 4.4. Determination of Total Phenolic Content (TPC)

In total, 50 μL of the methanolic extract (10 mg/mL) was mixed with 1 mL of distilled water, 0.5 mL of 0.2 N Folin-Ciocalteu reagent, and 2.5 mL of 5% sodium carbonate, then the sample was allowed to react in the darkness for 40 min at room temperature (20°C). The absorbance was read at 725 nm using a UV-Vis Genesys 150 Thermo Scientific spectrophotometer. A standard curve was made with a gallic acid solution (50 μg/mL) at concentrations of 10, 20, 30, 40, and 50 μg/mL. The results are presented in mg equivalent to gallic acid per g of methanolic extract (mg GAE/g of extract) [28].

### 4.5. Determination of Total Flavonoids

In total, 0.5 mL of the extract (10 mg/mL) was mixed with 1 mL with distilled water and 0.15 mL of 5% sodium nitrite; 5 min later, 0.15 mL of 10% aluminum chloride was added, then at 6 min, 2 mL of 4% sodium hydroxide was added. The sample was made up to 5 mL with distilled water, mixed, and allowed to react in the darkness for 15 min at room temperature. The absorbance was read at 510 nm against a blank. A standard curve was made with quercetin (200 μg/mL) at concentrations of 40, 80, 120, 160, and 200 μg/mL. The flavonoid content is presented as mg equivalent to quercetin per g of dry methanolic extract (mg QE/g of extract) [29].

### 4.6. Determination of the Antioxidant Capacity by the Free Radical Sequestration Method with 2,2-diphenyl-1-picrylhydrazyl 

For this assay, 150 μL of extract (10 mg/mL) was mixed with 2850 μL of a methanolic solution of DPPH radicals (20 mg/L) with the absorbance adjusted to 0.6 ± 0.02 nm. After mixing, the sample was incubated in the dark for 30 min and the absorbance was read at 515 nm. The standard curve was elaborated with Trolox at concentrations of 0 to 800 μmol/mL [30]. The antioxidant capacity equivalent to Trolox (TEAC) was calculated with the following formula:

$$TEAC\frac{\mu \mathrm{mol}TROLOX}{\mathrm{mg}\;ME}=IC_{50}TROLOX\;\left(\frac{\mu \mathrm{mol}}{\mathrm{mL}}\right)/IC_{50}sample\left(\frac{\mathrm{mg}}{\mathrm{mL}}\right)$$

To calculate the half inhibitory concentration (IC_50_), the percentage of inhibition of the DPPH radical was determined at concentrations of 5, 10, and 20 mg/mL of methanolic extract according to the following equation:

$$\%\;inhibiton\;of\;the\;DPPH\;radical=\frac{abs_{control}-abs_{sample}}{abs_{control}}\times 100$$

where *abs_control_* is the absorbance of the control without the sample at t = 0 min, and *abs_sample_* is the absorbance of the sample at t = 30 min.

### 4.7. Determination of the Antioxidant Capacity by the Sequestration Method with the Radical Cation of the 2.2′-azinobis-(3-ethylbenzothiazoline)-6-sulfonic acid

A standard solution (ST) was prepared by mixing 10 mL of ABTS (4.06 mg/mL) with 10 mL of potassium persulfate (0.7 mg/mL) and reacted for 12 h. The working solution (ST) was prepared with 1 mL of each extract and 60 mL of methanol. The absorbance was adjusted to 0.7 ± 0.02 with methanol at a wavelength of 734 nm, then 150 μL of the extract (5 mg/mL) was mixed with 2850 μL of the extract solution and incubated in the dark for 7 min, followed by reading the absorbances at 734 nm [31]. The standard curve was made with Trolox at 0–400 μmol/mL. The antioxidant capacity equivalent to Trolox (TEAC) was expressed as µmol ET/mg of the extract.

$$TEAC\frac{\mu \mathrm{mol}TROLOX}{\mathrm{mg}\;ME}=IC_{50}TROLOX\;\left(\frac{\mu \mathrm{mol}}{\mathrm{mL}}\right)/IC_{50}sample\left(\frac{\mathrm{mg}}{\mathrm{mL}}\right)$$

To calculate the half inhibitory concentrations (*IC_50_*), the percentage of inhibition of the ABTS radical was determined at concentrations of 1, 5, and 10 mg/mL as follows:

$$\%\;inhibiton\;of\;the\;ABTS\;radical\;=\frac{abs_{control}-abs_{sample}}{abs_{control}}\times 100$$

where *abs_control_* is the absorbance of the control without the sample at t = 0 min and *abs_sample_* is the absorbance of the sample at t = 7 min.

### 4.8. Phytochemical Analysis by LC-ESI-MS/MS of the Main Constituents of Methanolic Extracts of the Sprouts and Seeds of C. quinoa (Pasankalla Variety)

#### 4.8.1. Preparation of the Sample

The methanolic extracts of the sprouts and seeds of *C. quinoa* were weighed and diluted with methanol until a final concentration of 2 mg/mL had been obtained. Next, each sample was vortexed for 1 min and subsequently centrifuged for 10 min at 10,000 rpm. Finally, 800 µL of the 1 mg/mL solution supernatant (methanol:water, 1:1) was removed in vials for LC-MS analysis in a Dionex UltiMate 3000 liquid chromatograph (Thermo Fisher Scientific, San José, CA, USA) coupled to a Thermo QExactiveTM Plus Orbitrap mass spectrometer (Thermo Fisher Scientific, Bremen, Germany) with an electrospray ionization source. 

#### 4.8.2. Chromatographic Conditions

This analysis used a chromatographic column XBridge^®^ Amide BEH water (150 mm × 4.6 mm × 3.5 µm). Solvent A was 0.1% formic acid in water and Solvent B was 0.1% formic acid in ACN. The gradient elution of the method was as follows: 0–2 min, B 95%; 2–17.0 min, B 50%; 17–20.0 min, B 50%; 20.0–21.0 min, B 95%; 21.0–27.0 min, B 95%. The flow rate was 500 µL min^−1^ with injection of 8 µL and a column oven temperature of 40 °C.

#### 4.8.3. Mass Spectrometry Conditions

A full scan experiment combined with a fragmentation experiment (MS/MS) was performed for both electrospray ionization modes (ESI + and −). The ESI source parameters were as follows: spraying voltage: 3.9 kV (+) and 3.6 kV (−); envelope gas flow rate: 50 (arbitrary values); auxiliary gas flow: 10 (arbitrary values); tube lens voltage: 50 V; probe heater temperature: 400 °C; capillary temperature: 300 °C.

1. (. ESI +) mode: full MS mode parameters: 35,000 resolution; ACG target (automatic gain control): 5e5; maximum IT (injection time): 100 ms; scan range: 100–1200 m/z. 

Dd-MS^2^ (data-dependent acquisition experiment, DDA) mode parameters: 17,500 resolution; ACG objective: 1e5; maximum IT: 50 ms; loop count, 3; isolation window: 1–2 m/z; topN, 3; NCE (stepped normalized collision energy): 15, 30, and 40.

2. (. ESI −) mode: full MS mode parameters: 35,000 resolution; ACG objective: 5e5; maximum IT: 100 ms; range, 100–1200 m/z.

Dd-MS^2^ (data-dependent acquisition experiment, DDA) mode parameters: 17,500 resolution; ACG objective: 1e5; maximum IT: 50 ms; loop count, 3; isolation window: 1–2 m/z; topN: 3; NCE: 15, 20, and 40.

Data acquisition and processing were performed with Thermo XcaliburTM software version 3.0 (Thermo Fisher Scientific Inc., Waltham, MA, USA) with the Qual Browser, and metabolite annotations were performed with MS-Dial software version 4.70 (Riken, Osaka University, Suita City, Japan) using the MS-Dial metabolomics MPS spectral kit library (available at: http://prime.psc.riken.jp/compms/msdial/main.html; last updated on 13 April 2021).

### 4.9. Data Analysis

The results are presented as the means plus standard deviation of three repetitions. The differences between the means were analyzed using paired sample t-test for total phenols, flavonoids, antioxidant capacity, and the half inhibitory concentration (IC_50_), using SPSS software. Pearson’s correlation coefficient was determined to establish the relationships among total phenols and flavonoids, antioxidant capacity (TEAC), and the half inhibitory concentration (*IC_50_*), with a *p*-value less than 0.05 being significant.

## 5. Conclusions

Based on our results, we concluded that quinoa sprouts germinated for 72 h had higher total phenolic content and total flavonoids compared with seed extracts, and these correlated with its high antioxidant capacity. Furthermore, sprout extracts had better IC_50_ and TEAC values in the DPPH and ABTS assays. The best variety of quinoa was Pasankalla, which showed a high antioxidant capacity and also contained 90 and 93 phytochemical constituents in the sprout and seed extract, respectively. Some chemical groups highlighted were amino acids, organic acids, phenolic acids, flavonoids, fatty acids, lipids, saponins, and sugars, with a greater diversity of essential amino acids found in sprouts than in seeds.

## Figures and Tables

**Table 1:** Total phenolic content (TPC) and total flavonoids (TF) in the sprouts and seeds of 20 varieties of quinoa.

Variety	TPC mg EAG/g ME		TF mg EQ/g ME	
	Quinoa Sprouts Mean ± SD	Quinoa Seeds Mean ± SD	Quinoa Sprouts Mean ± SD	Quinoa Seeds Mean ± SD
White Junín Ayacucho	23.32 ± 1.63	20.95 ± 0.79	11.52 ± 0.26	8.77 ± 0.26 *
2. T-256	24.78 ± 0.21	13.82 ± 1.04 *	11.23 ± 0.19	10.23 ± 0.95
3. Pasankalla	31.28 ± 0.42	28.32 ± 0.49 *	13.48 ± 0.38	11.52 ± 0.92 *
4. Suano Puno	19.62 ± 0.42	17.25 ± 0.66 *	8.60 ± 0.48	8.56 ± 0.38
5. T-38	21.05 ± 0.40	21.75 ± 1.25	10.06 ± 0.57	9.81 ± 0.25
6. Yellow Sacaca	24.22 ± 0.31	23.58 ± 0.61	11.19 ± 0.38	8.23 ± 0.29 *
7. T-45	21.02 ± 0.15	19.38 ± 2.06	11.06 ± 0.21	8.39 ± 0.38 *
8. Santa Ana	23.02 ± 0.74	18.23 ± 1.01 *	9.94 ± 0.63	7.06 ± 0.33 *
9. T-61 Pomata	21.12 ± 1.50	15.55 ± 0.20 *	10.94 ± 0.33	8.73 ± 0.31 *
10. CQA-048	28.82 ± 0.67	21.32 ± 0.72 *	7.44 ± 0.50	6.23 ± 0.26 *
11. Black Collana	28.58 ± 1.21	26.98 ± 0.25 *	13.44 ± 0.58	8.73 ± 0.14 *
12. T-72 Huancayo	19.15 ± 1.54	18.58 ± 0.65	12.35 ± 0.48	9.81 ± 0.45 *
13. CQA-043	26.05 ± 0.17	11.72 ± 0.32 *	12.15 ± 0.08	11.31 ± 0.50
14. Salcedo	20.98 ± 1.99	12.38 ± 0.61 *	11.94 ± 0.13	9.81 ± 0.45 *
15. Ayacucho Compuesto	28.05 ± 0.53	21.42 ± 1.17 *	11.19 ± 0.25	10.98 ± 0.40
16. White Choclito	24.02 ± 0.78	20.78 ± 1.86	11.52 ± 0.31	9.90 ± 0.26 *
17. Red	26.05 ± 0.36	20.45 ± 0.44 *	12.31 ± 0.50	10.52 ± 0.19 *
18. Yellow Maranganí	27.98 ± 0.70	22.82 ± 1.12 *	13.52 ± 0.44	10.98 ± 0.52 *
19. Black Coito	28.18 ± 0.35	24.42 ± 0.75 *	14.31 ± 0.50	9.94 ± 0.13 *
20. Black	24.12 ± 0.64	20.78 ± 0.35 *	12.31 ± 0.45	9.73 ± 0.38 *
Total Average ± SD	24.57 ± 3.49	20.12 ± 4.37 *	11.52 ± 1.67	9.46 ± 1.40 *

* *p* < 0.05 (paired sample *t*-test); mg GAE/g ME: mg equivalent to gallic acid per g of methanolic extract.; mg EQ/g ME: mg equivalent to quercetin per g of methanolic extract.

**Table 2:** Antioxidant capacity equivalent to Trolox (TEAC) of the radical DPPH and ABTS of methanolic extracts of sprouts and seeds of 20 varieties of quinoa.

Variety	TEAC-DPPH µmol TE/mg MS		TEAC-ABTS µmol TE/mg MS	
	Quinoa Sprouts Mean ± SD	Quinoa Seeds Mean ± SD	Quinoa Sprouts Mean ± SD	Quinoa Seeds Mean ± SD
White Junín Ayacucho	31.26 ± 0.56	28.47 ± 1.44	64.78 ± 1.63	54.68 ± 0.48 *
2. T-256	28.38 ± 0.27	25.88 ± 0.72 *	62.84 ± 1.65	61.95 ± 0.96
3. Pasankalla	37.65 ± 0.88	29.60 ± 0.54 *	78.79 ± 0.86	54.19 ± 0.41 *
4. Suano Puno	25.90 ± 0.36	25.24 ± 0.22	78.66 ± 2.02	53.10 ± 1.03 *
5. T-38	27.67 ± 0.30	25.03 ± 0.18 *	59.96 ± 5.62	48.88 ± 1.52 *
6. Yellow Sacaca	30.54 ± 1.17	29.37 ± 0.82	63.21 ± 0.60	58.38 ± 2.14 *
7. T-45	25.94 ± 0.29	25.54 ± 0.17 *	60.55 ± 3.46	45.80 ± 0.37 *
8. Santa Ana	28.08 ± 0.07	26.93 ± 0.23 *	60.74 ± 1.06	53.66 ± 0.55 *
9. T-61 Pomata	25.92 ± 0.15	25.77 ± 0.15 *	65.40 ± 0.96	50.56 ± 2.29 *
10. CQA-048	26.32 ± 0.12	25.64 ± 0.17	62.29 ± 2.59	64.91 ± 5.06
11. Black Collana	29.26 ± 0.40	25.90 ± 0.15 *	90.84 ± 2.22	60.56 ± 4.28 *
12. T-72 Huancayo	26.97 ± 0.40	25.75 ± 0.20	68.67 ± 0.64	59.40 ± 0.09 *
13. CQA-043	26.17 ± 0.55	25.89 ± 0.23	57.05 ± 2.62	56.35 ± 0.34
14. Salcedo	26.21 ± 0.24	25.96 ± 0.23 *	64.95 ± 0.83	58.21 ± 0.19 *
15. Ayacucho Compuesto	26.45 ± 0.27	26.26 ± 0.20	68.91 ± 0.61	65.74 ± 0.25 *
16. White Choclito	27.30 ± 0.23	26.88 ± 0.31 *	58.84 ± 2.73	57.92 ± 0.75
17. Red	28.60 ± 0.20	26.93 ± 0.36 *	75.79 ± 1.26	67.04 ± 0.79 *
18. Yellow Maranganí	27.51 ± 0.29	26.20 ± 0.12 *	78.11 ± 1.69	63.76 ± 0.70 *
19. Black Coito	28.04 ± 0.10	26.56 ± 0.16 *	69.41 ± 0.87	63.68 ± 0.93 *
20. Black	27.67 ± 0.25	26.09 ± 0.06 *	78.79 ± 2.36	56.43 ± 0.52 *
Total Average ± SD	28.09 ± 2.68	26.50 ± 1.30 *	68.43 ± 8.96	57.71 ± 5.83 *

* *p* < 0.05; paired sample *t*-test.

**Table 3:** Half inhibitory concentration (IC50) of the radicals DPPH and ABTS of methanolic extracts of sprouts and seeds of 20 varieties of quinoa.

Variety	IC_50_ (mg/mL)			
	DPPH		ABTS	
	Quinoa Sprouts Mean ± SD	Quinoa Seeds Mean ± SD	Quinoa Sprouts Mean ± SD	Quinoa Seeds Mean ± SD
White Junín Ayacucho	15.29 ± 0.27	16.81 ± 0.83	4.27 ± 0.11	5.06 ± 0.05 *
2. T-256	16.84 ± 0.16	18.47 ± 0.52 *	4.40 ± 0.12	4.47 ± 0.07
3. Pasankalla	12.69 ± 0.29	16.15 ± 0.30 *	3.51 ± 0.04	5.11 ± 0.04 *
4. Suano Puno	18.45 ± 0.26	18.93 ± 0.16	3.52 ± 0.10	5.21 ± 0.10 *
5. T-38	17.27 ± 0.19	19.10 ± 0.14 *	4.64 ± 0.45	5.66 ± 0.18 *
6. Yellow Sacaca	15.66 ± 0.60	16.28 ± 0.46	4.38 ± 0.04	4.74 ± 0.17 *
7. T-45	18.42 ± 0.20	18.71 ± 0.12 *	4.58 ± 0.26	6.04 ± 0.05 *
8. Santa Ana	17.02 ± 0.04	17.75 ± 0.14 *	4.55 ± 0.08	5.16 ± 0.06 *
9. T-61 Pomata	18.43 ± 0.08	18.54 ± 0.11 *	4.23 ± 0.06	5.48 ± 0.25 *
10. CQA-048	18.15 ± 0.09	18.63 ± 0.12	4.45 ± 0.18	4.28 ± 0.32
11. Black Collana	16.33 ± 0.22	18.45 ± 0.10 *	3.05 ± 0.08	4.58 ± 0.32 *
12. T-72 Huancayo	17.72 ± 0.27	18.55 ± 0.14	4.03 ± 0.04	4.66 ± 0.01 *
13. CQA-043	18.26 ± 0.39	18.46 ± 0.17	4.86 ± 0.22	4.91 ± 0.03
14. Salcedo	18.23 ± 0.17	18.40 ± 0.16 *	4.26 ± 0.06	4.75 ± 0.02 *
15. Ayacucho Compuesto	18.07 ± 0.18	18.20 ± 0.14	4.01 ± 0.04	4.21 ± 0.02 *
16. White Choclito	17.51 ± 0.15	17.34 ± 0.20	4.71 ± 0.21	4.78 ± 0.06
17. Red	16.71 ± 0.12	17.75 ± 0.23 *	3.95 ± 0.29	4.13 ± 0.05
18. Yellow Maranganí	17.37 ± 0.19	18.24 ± 0.09 *	3.54 ± 0.07	4.34 ± 0.05 *
19. Black Coito	17.04 ± 0.06	17.99 ± 0.11 *	3.99 ± 0.05	4.41 ± 0.06 *
20. Black	17.27 ± 0.15	18.31 ± 0.04 *	3.51 ± 0.11	4.90 ± 0.05
Total Average ± SD	17.14 ± 1.37	18.05 ± 0.84 *	4.12 ± 0.50	4.84 ± 0.51

* *p* < 0.05; paired sample *t*-test.

**Table 4:** Pearson’s correlation coefficients among total phenols, total flavonoids, antioxidant capacity (TEAC), and the half inhibitory concentration (IC50) of the radicals DPPH and ABTS in sprouts and seeds of quinoa.

Correlations		TEAC-DPPH	TEAC-ABTS	IC_50_ DPPH	IC_50_ ABTS
TPC of quinoa seeds	Pearson’s correlation	0.480 **	0.352 **	−0.477 **	−0.331 **
	*p*-value	<0.0001	0.006	<0.0001	0.010
TF of quinoa sprouts	Pearson’s correlation	0.372 **	0.407 **	−0.393 **	−0.404 **
	*p*-value	0.003	0.001	0.002	0.001
TPC of quinoa sprouts	Pearson’s correlation	0.436 **	0.106	−0.433 **	−0.087
	*p*-value	<0.0001	0.421	0.001	0.508
TF of quinoa seeds	Pearson’s correlation	0.092	0.202	−0.098	−0.214
	*p*-value	0.483	0.121	0.455	0.100

** The correlation is significant at the 0.01 level (bilateral).

**Table 5:** Number of annotated metabolites (via MS and MS/MS) in each extract according to the ESI (−) and ESI (+) ionization modes.

C. quinoa (Pasankalla Variety)	ESI (−)	ESI (+)	ESI (+/−)	Total
Seeds	58	28	7	93
Sprouts	45	33	12	90

**Table 6:** Phytochemical constituents of quinoa sprouts (Pasankalla variety) determined by LC-ESI-MS/MS.

#	Rt (min)	Theoretical Mass (Neutral Form)	Molecular Formula (Neutral Form)	Predicted Metabolite	Chemical Group
1	3.85	340.1885884	C_18_H_28_O_6_	[5-acetyloxy-3-(hydroxymethyl)-2-oxo-6-propan-2-ylcyclohex-3-en-1-yl] 3-methylpentanoate	Menthane monoterpenoids
2	3.91	130.0266086	C_5_H_6_O_4_	Citraconic acid	Organic acids
3	4.30	313.131408	C_18_H_19_NO_4_	Feruloyl tyramine	Ferulic acid and derivatives
4	4.40	138.031694	C_7_H_6_O_3_	Salicylic acid	Salicylic acids
5	4.64	146.0579087	C_6_H_10_O_4_	2-Methylglutaric acid	Methyl-branched fatty acids
6	4.69	311.1157579	C_18_H_17_NO_4_	Feruloyl dehydrotyramine	Ferulic acid and derivatives
7	4.75	132.0422586	C_5_H_8_O_4_	Glutaric acid	Dicarboxylic acids and derivatives
8	5.12	118.0266086	C_4_H_6_O_4_	Succinic acid (Isomer I)	Dicarboxylic acids and derivatives
9	5.35	173.1051933	C_8_H_15_NO_3_	n-Acetyl-L-leucine (Isomer I)	Leucine and derivatives
10	5.77	118.0266086	C_4_H_6_O_4_	Succinic acid (Isomer II)	Dicarboxylic acids and derivatives
11	5.79	162.0528233	C_6_H_10_O_5_	β-hydroxy-β-methylglutaric acid (Isomer I)	Hydroxy fatty acids
12	5.87	123.0320284	C_6_H_5_NO_2_	Isonicotinic acid	Pyridinecarboxylic acids
13	6.03	154.0266086	C_7_H_6_O_4_	2,3-Dihydroxybenzoic acid	Salicylic acids
14	6.24	173.1051933	C_8_H_15_NO_3_	n-Acetyl-L-leucine (Isomer II)	Leucine and derivatives
15	6.31	162.0528233	C_6_H_10_O_5_	β-hydroxy-β-methylglutaric acid (Isomer II)	Hydroxy fatty acids
16	6.68	219.1106725	C_9_H_17_NO_5_	Pantothenic acid (Isomer I)	Vitamin B5
	6.69	219.1106725	C_9_H_17_NO_5_		
17	7.09	219.1106725	C_9_H_17_NO_5_	Pantothenic acid (Isomer II)	Vitamin B5
	7.12	219.1106725	C_9_H_17_NO_5_		
18	7.84	298.1568945	C_19_H_22_O_3_	Aurapten	Coumarins
19	7.86	129.042593	C_5_H_7_NO_3_	L-Pyroglutamic acid	Alpha amino acids and derivatives
20	10.07	480.3087035	C_27_H_44_O_7_	NCGC00168839-02!(2S,3R,5R,10R,13R,14S,17S)-2,3,14-trihydroxy-10,13-dimethyl-17-[(2R,3R)-2,3,6-trihydroxy-6-methylheptan-2-yl]-2,3,4,5,9,11,12,15,16,17-decahydro-1H-cyclopenta[a]phenanthren-6-one Syn. Phytoecdysteroids	Phytoecdysteroids
	10.08	480.3087035	C_27_H_44_O_7_		
21	10.52	648.3873477	C_36_H_56_O_10_	(2S,3S,4S,5R,6R)-6-[[(3S,6aR,6bS,8aS,14bR)-8a-carboxy-4-(hydroxymethyl)-4,6a,6b,11,11,14b-hexamethyl-1,2,3,4a,5,6,7,8,9,10,12,12a,14,14a-tetradecahydropicen-3-yl]oxy]-3,4,5-trihydroxyoxane-2-carboxylic acid Syn. NCGC00381031-01_C36H56O10_Olean-12-en-28-oic acid, 3-(beta-D-glucopyranuronosyloxy)-23-hydroxy-, (3beta,5xi,9xi,18xi)-	Triterpene saponins
22	10.91	372.1420321	C_17_H_24_O_9_	Syringin	Phenolic glycosides
23	11.06	356.110732	C_16_H_20_O_9_	NCGC00180844-02!(E)-3-[4-methoxy-2-[(2S,3R,4S,5S,6R)-3,4,5-trihydroxy-6-(hydroxymethyl)oxan-2-yl]oxyphenyl]prop-2-enoic acid Syn. 2-O-Glucosyloxy-4-methoxycinnamic acid	Phenolic glycosides
24	11.50	244.069536	C_9_H_12_N_2_O_6_	Uridine	Pyrimidine nucleosides
25	12.30	810.440171	C_42_H_66_O_15_	NCGC00347541-02_C42H66O15_beta-D-Glucopyranose, 1-O-[(3beta,5xi,9xi,18xi)-3-(beta-D-glucopyranuronosyloxy)-29-hydroxy-28-oxoolean-12-en-28-yl]-	Triterpene saponins
26	12.37	477.285539	C_23_H_44_NO_7_P	Lysophosphatidylethanolamine LPE 18:2	Lipids
27	12.42	453.285539	C_21_H_44_NO_7_P	Lysophosphatidylethanolamine LPE 16:0	Lipids
	12.43	453.285539	C_21_H_44_NO_7_P		
28	12.44	152.0334253	C_5_H_4_N_4_O_2_	Xanthine (Isomer I)	Xanthines
28	12.86	519.3324892	C_26_H_50_NO_7_P	Lysophosphatidylcholine LPC 18:2	Lipids
29	12.89	495.3324892	C_24_H_50_NO_7_P	Lysophosphatidylcholine LPC 16:0	Lipids
	12.91	495.3324892	C_24_H_50_NO_7_P		
30	12.93	131.0946286	C_6_H_13_NO_2_	Alanine betaine	Alanine and derivatives
31	12.93	517.3168391	C_26_H_48_NO_7_P	Lysophosphatidylcholine LPC 18:3	Lipids
	12.94	517.3168391	C_26_H_48_NO_7_P		
32	12.99	956.4980797	C_48_H_76_O_19_	6-[[(3S,6aR,6bS,8aS,14bR)-4,4,6a,6b,11,11,14b-Heptamethyl-8a-[3,4,5-trihydroxy-6-(hydroxymethyl)oxan-2-yl]oxycarbonyl-1,2,3,4a,5,6,7,8,9,10,12,12a,14,14a-tetradecahydropicen-3-yl]oxy]-3,5-dihydroxy-4-[3,4,5-trihydroxy-6-(hydroxymethyl)oxan-2-yl]oxyoxane-2-carboxylic acid Syn. NCGC00385168-01_C48H76O19_Hexopyranose, 1-O-[(3beta,5xi,9xi,18xi)-3-[(3-O-hexopyranosylhexopyranuronosyl)oxy]-28-oxoolean-12-en-28-yl]-	Triterpene saponins
33	13.20	315.2773439	C_18_H_37_NO_3_	Dehydrophytosphingosine	Lipids
34	13.39	456.3603452	C_30_H_48_O_3_	Ursolic acid Syn. Isomer I	Triterpenoids
35	13.40	956.4980797	C_48_H_76_O_19_	6-[[(3S,6aR,6bS,8aS,14bR)-4,4,6a,6b,11,11,14b-Heptamethyl-8a-[3,4,5-trihydroxy-6-(hydroxymethyl)oxan-2-yl]oxycarbonyl-1,2,3,4a,5,6,7,8,9,10,12,12a,14,14a-tetradecahydropicen-3-yl]oxy]-3,5-dihydroxy-4-[3,4,5-trihydroxy-6-(hydroxymethyl)oxan-2-yl]oxyoxane-2-carboxylic acidSyn. NCGC00385168-01_C48H76O19_Hexopyranose, 1-O-[(3beta,5xi,9xi,18xi)-3-[(3-O-hexopyranosylhexopyranuronosyl)oxy]-28-oxoolean-12-en-28-yl]-	Triterpene saponins
36	13.49	152.0684734	C_5_H_12_O_5_	Xylitol (Isomer I)	Sugar alcohols
37	13.60	291.0954163	C_11_H_17_NO_8_	N-fructosyl pyroglutamate	N-fructosyl amino acids
38	13.63	284.075684	C_10_H_12_N4O_6_	Xanthosine	Purine nucleosides
	13.64	284.075684	C_10_H_12_N_4_O_6_		
39	13.64	152.0334253	C_5_H_4_N_4_O_2_	Xanthine (Isomer II)	Xanthines
40	13.72	152.0684734	C_5_H_12_O_5_	Xylitol (Isomer II)	Sugar alcohols
41	13.80	454.3446952	C_30_H_46_O_3_	NCGC00380944-01_C30H46O3_(3beta,5xi,9xi,13alpha,17alpha,18xi)-3-Hydroxy-13,28-epoxyurs-11-en-28-oneSyn. 3-Hydroxy-11-ursen-28,13-olide	Triterpenoids
42	13.98	456.3603452	C_30_H_48_O_3_	Ursolic acid (Isomer II)	Triterpenoids
43	14.34	267.0967538	C_10_H_13_N_5_O_4_	Adenosine	Purine nucleosides
44	14.45	180.063388	C_6_H_12_O_6_	Psicose	Monosaccharides
45	14.69	456.3603452	C_30_H_48_O_3_	Ursolic acid (Isomer III)	Triterpenoids
46	14.80	120.0575148	C_8_H_8_O	Phenylacetaldehyde	Phenylacetaldehydes
47	14.80	165.0789785	C_9_H_11_NO_2_	Phenylalanine	Amino acids
48	14.92	204.0898776	C_11_H_12_N_2_O_2_	Tryptophan	Amino acids
49	15.03	131.0946286	C_6_H_13_NO_2_	Isoleucine	Isoleucine and derivatives
50	15.11	756.1901639	C_36_H_36_O_18_	NCGC00381212-01![6-[2-(3,4-dihydroxyphenyl)-5,7-dihydroxy-4-oxochromen-3-yl]oxy-4,5-dihydroxy-2-[(3,4,5-trihydroxy-6-methyloxan-2-yl)oxymethyl]oxan-3-yl] (E)-3-(4-hydroxyphenyl)prop-2-enoate	Flavonoid-O-glycosides
51	15.14	756.2112932	C_33_H_40_O_20_	2-(3,4-Dihydroxyphenyl)-3-[(2S,3R,4S,5S,6R)-4,5-dihydroxy-3-[(2R,3R,4R,5R,6S)-3,4,5-trihydroxy-6-methyloxan-2-yl]oxy-6-[[(2R,3R,4R,5R,6S)-3,4,5-trihydroxy-6-methyloxan-2-yl]oxymethyl]oxan-2-yl]oxy-5,7-dihydroxychromen-4-oneSyn. Quercetin 3-O-rutinoside-(1-2)-O-rhamnoside	Flavonoid-O-glycosides
52	15.21	182.079038	C_6_H_14_O_6_	D-sorbitol	Sugar alcohols
53	15.31	117.0789785	C_5_H_11_NO_2_	Betaine	Alpha amino acids
54	15.39	180.063388	C_6_H_12_O_6_	Mannose (Isomer I)	Hexoses
55	15.41	136.0371732	C_4_H_8_O_5_	Threonic acid (Isomer I)	Sugar acids and derivatives
56	15.41	150.0528233	C_5_H_10_O_5_	Xylose	Pentoses
57	15.57	104.107539	C_5_H_14_NO	Choline	Cholines
58	15.61	136.0371732	C_4_H_8_O_5_	Threonic acid (Isomer II)	Sugar acids and derivatives
59	15.63	180.063388	C_6_H_12_O_6_	Mannose(Isomer II)	Hexoses
60	15.70	196.0583026	C_6_H_12_O_7_	D-gluconic acid (Isomer I)	Medium-chain hydroxy acids and derivatives
61	15.71	137.0476784	C_7_H_7_NO_2_	Trigonelline	Alkaloids and derivatives
	15.82	137.0476784	C_7_H_7_NO_2_		
62	15.87	145.0851265	C_5_H_11_N_3_O_2_	4-Guanidinobutyric acid	Gamma amino acids and derivatives
63	15.98	196.0583026	C_6_H_12_O_7_	D-gluconic acid (Isomer II)	Medium-chain hydroxy acids and derivatives
64	15.96	181.0738931	C_9_H_11_NO_3_	Tyrosine	Tyrosine and derivatives
65	16.15	165.0789785	C_9_H_11_NO_2_	Phenylalanine	Phenylalanine and derivatives
66	16.16	212.0896027	C_7_H_16_O_7_	Volemitol	Sugar alcohols
67	16.45	293.1474519	C_12_H_23_NO_7_	N-fructosyl isoleucine	N-fructosyl amino acids
	16.48	293.1474519	C_12_H_23_NO_7_		
	16.54	293.1474519	C_12_H_23_NO_7_		
68	16.62	103.0633285	C_4_H_9_NO_2_	4-Aminobutyric acid Syn. 4-Aminobutanoic acid/GABA	Gamma amino acids and derivatives
69	17.15	147.0531577	C_5_H_9_NO_4_	L-Glutamic acid (Isomer I)	Glutamic acid and derivatives
70	17.18	342.1162113	C_12_H_22_O_11_	Melibiose (Isomer I)	O-glycosyl compounds
71	17.25	342.1162113	C_12_H_22_O_11_	Isomaltulose	O-glycosyl compounds
72	17.26	342.1162113	C_12_H_22_O_11_	Trehalose	Disaccharide
73	17.27	129.042593	C_5_H_7_NO_3_	Pyroglutamic acid	Alpha amino acids and derivatives
74	17.29	147.0531577	C_5_H_9_NO_4_	L-glutamic acid (Isomer II)	Glutamic acid and derivatives
75	17.38	119.0582431	C_4_H_9_NO_3_	Threonine(Isomer I)	L-alpha-amino acids
76	17.46	119.0582431	C_4_H_9_NO_3_	Threonine (Isomer II)	L-alpha-amino acids
77	18.02	165.0459638	C_5_H_11_NO_3_S	Methioninesulfoxide	Alpha amino acids
78	18.02	342.1162113	C_12_H_22_O_11_	Melibiose (Isomer II)	O-glycosyl compounds
79	18.10	146.0691421	C_5_H_10_N_2_O_3_	Glutamine	D-alpha-amino acids
	18.14	146.0691421	C_5_H_10_N_2_O_3_		
80	18.12	105.042593	C_3_H_7_NO_3_	Serine	Serine and derivatives
	18.16	105.042593	C_3_H_7_NO_3_		
81	18.31	344.1318613	C_12_H_24_O_11_	Maltitol	Fatty acyl glycosides of mono- and disaccharides
82	18.40	132.053492	C_4_H_8_N_2_O_3_	Asparagine	Asparagine and derivatives
	18.41	132.053492	C_4_H_8_N_2_O_3_		
83	18.53	342.1162113	C_12_H_22_O_11_	Melibiose (Isomer III)	O-glycosyl compounds
84	18.84	504.1690346	C_18_H_32_O_16_	Melezitose (Isomer I)	Oligosaccharides
	18.85	504.1690346	C_18_H_32_O_16_		
	18.86	504.1690346	C_18_H_32_O_16_		
85	19.13	504.1690346	C_18_H_32_O_16_	Melezitose (Isomer II)	Oligosaccharides
86	19.14	504.1690346	C_18_H_32_O_16_	Maltotriose (Isomer I)	Oligosaccharides
87	19.33	504.1690346	C_18_H_32_O_16_	Maltotriose (Isomer II)	Oligosaccharides
88	19.34	504.1690346	C_18_H_32_O_16_	Raffinose	Oligosaccharides
89	21.88	155.0694765	C_6_H_9_N_3_O_2_	L-Histidine	Histidine and derivatives
90	22.28	174.1116756	C_6_H_14_N_4_O_2_	L-Arginine	L-alpha-amino acids

**Table 7:** Phytochemical constituents of quinoa seeds (Pasankalla variety) determined by LC-ESI-MS/MS.

	Rt (min)	Theoretical Mass (Neutral Form)	Molecular Formula (Neutral Form)	Predicted Metabolite	Chemical Group
1	3.19	340.1885884	C_18_H_28_O_6_	[5-Acetyloxy-3-(hydroxymethyl)-2-oxo-6-propan-2-ylcyclohex-3-en-1-yl] 3-methylpentanoate	Menthane monoterpenoids
2	3.72	145.0527638	C_9_H_7_NO	2-Hydroxyquinoline	Hydroquinolones
3	3.74	122.0367794	C_7_H_6_O_2_	3-Hydroxybenzaldehyde	Phenolic compounds
4	3.78	206.0579087	C_11_H_10_O_4_	Isoeugenitol	Chromones
5	3.80	152.047344	C_8_H_8_O_3_	4-Hydroxyphenylacetic acid	1-Hydroxy-2-unsubstituted benzenoids
6	3.85	168.0422586	C_8_H_8_O_4_	3,4-Dihydroxyphenylacetate (Isomer I) Syn. Homoprotocatechuic acid	Catechols
7	3.89	154.0266086	C_7_H_6_O_4_	Pyrocatechuic acid (Isomer I)	Salicylic acids
8	3.96	130.0266086	C_5_H_6_O_4_	Citraconic acid	Methyl-branched fatty acids
9	4.13	164.047344	C_9_H_8_O_3_	3-Hydroxycinnamic acid	Hydroxycinnamic acids
10	4.20	132.0786442	C_6_H_12_O_3_	2-Hydroxyisocaproic acid	Hydroxy fatty acids
11	4.20	160.0735588	C_7_H_12_O_4_	3-Methyladipic acid	Medium-chain fatty acids
12	4.22	168.0422586	C_8_H_8_O_4_	3,4-Dihydroxyphenylacetate (Isomer II) Syn. Homoprotocatechuic acid	Catechols
13	4.29	154.0266086	C_7_H_6_O_4_	Pyrocatechuic acid (Isomer II)	Salicylic acids
14	4.43	138.031694	C_7_H_6_O_3_	Salicylic acid	Salicylic acids
15	4.66	146.0579087	C_6_H_10_O_4_	2-Methylglutaric acid	Methyl-branched fatty acids
16	4.75	132.0422586	C_5_H_8_O_4_	Glutaric acid (Isomer I)	Dicarboxylic acids and derivatives
17	4.75	194.0579087	C_10_H_10_O_4_	*trans*-4-Hydroxy-3-methoxycinnamate (Isomer I)	Hydroxycinnamic acids
18	4.98	134.0215232	C_4_H_6_O_5_	Malic acid (Isomer I)	Beta hydroxy acids and derivatives
19	5.07	164.047344	C_9_H_8_O_3_	3-Hydroxycinnamic acid (Isomer I)	Hydroxycinnamic acids
20	5.14	118.0266086	C_4_H_6_O_4_	Succinic acid (Isomer I)	Dicarboxylic acids and derivatives
21	5.45	132.0422586	C_5_H_8_O_4_	Glutaric acid (Isomer II)	Dicarboxylic acids and derivatives
22	5.75	118.0266086	C_4_H_6_O_4_	Succinic acid (Isomer II)	Dicarboxylic acids and derivatives
23	5.82	194.0579087	C_10_H_10_O_4_	*trans*-4-Hydroxy-3-methoxycinnamate (Isomer II)	Hydroxycinnamic acids
24	5.83	123.0320284	C_6_H_5_NO_2_	Isonicotinic acid	Pyridinecarboxylic acids
25	5.98	110.0367794	C_6_H_6_O_2_	Catechol	Catechols
26	6.26	134.0215232	C_4_H_6_O_5_	Malic acid (Isomer II)	Beta hydroxy acids and derivatives
27	6.26	164.047344	C_9_H_8_O_3_	3-Hydroxycinnamic acid (Isomer II)	Hydroxycinnamic acids
28	7.05	219.1106725	C_9_H_17_NO_5_	Pantothenic acid	Secondary alcohols
	7.11	219.1106725	C_9_H_17_NO_5_		
29	7.27	516.3298328	C_27_H_48_O_9_	MGMG 18:2	Lipids
30	7.40	122.0480128	C_6_H_6_N_2_O	Nicotinamide	Nicotinamides
31	7.82	264.1110069	C_13_H_16_N_2_O_4_	Phenylacetylglutamine	N-acyl-alpha amino acids
32	7.83	129.042593	C_5_H_7_NO_3_	5-Oxo-D-proline Syn. D-Pyroglutamic acid	Proline and derivatives
33	8.38	514.3141828	C_27_H_46_O_9_	NCGC00380867-01_C27H46O9_9,12,15-Octadecatrienoic acid, 3-(hexopyranosyloxy)-2-hydroxypropyl ester, (9Z,12Z,15Z)-	Glycosylmonoacylglycerols
34	9.53	494.3243536	C_28_H_46_O_7_	NCGC00169545-02!(2S,3R,5R,10R,13R,14S,17S)-2,3,14-trihydroxy-10,13-dimethyl-17-[(2R,3R,5R)-2,3,6-trihydroxy-5,6-dimethylheptan-2-yl]-2,3,4,5,9,11,12,15,16,17-decahydro-1H-cyclopenta[a]phenanthren-6-one Syn. Makisterone A	Phytoecdysteroids
35	10.03	480.3087035	C_27_H_44_O_7_	NCGC00168839-02!(2S,3R,5R,10R,13R,14S,17S)-2,3,14-trihydroxy-10,13-dimethyl-17-[(2R,3R)-2,3,6-trihydroxy-6-methylheptan-2-yl]-2,3,4,5,9,11,12,15,16,17-decahydro-1H-cyclopenta[a]phenanthren-6-one Syn. Ecdysterone	Phytoecdysteroids
	10.04	480.3087035	C_27_H_44_O_7_		
40	10.06	722.5097847	C_38_H_75_O_10_P	[3-[[2,3-Dihydroxypropoxy]-hydroxyphosphoryl]oxy-2-hexadecanoyloxypropyl] hexadecanoate Syn. Dipalmitoylphosphatidylglycerol	Phosphatidylglycerol
41	10.90	372.1420321	C_17_H_24_O_9_	Syringin	Phenolic glycosides
42	10.96	478.0747403	C_21_H_18_O_13_	Quercetin-3-glucuronide	Flavonoid-O-glucuronides
43	11.49	244.069536	C_9_H_12_N_2_O_6_	Uridine	Pyrimidine nucleosides
44	12.03	281.1124038	C_11_H_15_N_5_O_4_	2′-O-methyladenosine	Purine nucleosides
45	12.36	477.285539	C_23_H_44_NO_7_P	Lysophosphatidylethanolamine LPE 18:2	Lipids
46	12.40	453.285539	C_21_H_44_NO_7_P	Lysophosphatidylethanolamine LPE 16:0	Lipids
47	12.59	131.0946286	C_6_H_13_NO_2_	Alanine betaine	Alanine and derivatives
48	12.76	521.3481393	C_26_H_52_NO_7_P	Lysophosphatidylcholine LPC 18:1	Lipids
	12.78	521.3481393	C_26_H_52_NO_7_P		
49	12.85	519.3324892	C_26_H_50_NO_7_P	Lysophosphatidylcholine LPC 18:2	Lipids
50	12.88	152.0684734	C_5_H_12_O_5_	L-arabitol (Isomer I)	Sugar alcohols
51	12.89	495.3324892	C_24_H_50_NO_7_P	Lysophosphatidylcholine LPC 16:0	Lipids
	12.90	495.3324892	C_24_H_50_NO_7_P		
52	12.91	517.3168391	C_26_H_48_NO_7_P	Lysophosphatidylcholine LPC 18:3	Lipids
	12.95	517.3168391	C_26_H_48_NO_7_P		
53	13.11	639.3383623	C_29_H_54_NO_12_P	Hexosyl LPE 18:2	Lipids
54	13.16	315.2773439	C_18_H_37_NO_3_	Dehydrophytosphingosine Syn. 4-Hydroxy-8-sphingenine	Lipids
55	13.43	956.4980797	C_48_H_76_O_19_	6-[[(3S,6aR,6bS,8aS,14bR)-4,4,6a,6b,11,11,14b-heptamethyl-8a-[3,4,5-trihydroxy-6-(hydroxymethyl)oxan-2-yl]oxycarbonyl-1,2,3,4a,5,6,7,8,9,10,12,12a,14,14a-tetradecahydropicen-3-yl]oxy]-3,5-dihydroxy-4-[3,4,5-trihydroxy-6-(hydroxymethyl)oxan-2-yl]oxyoxane-2-carboxylic acid Syn. NCGC00385168-01_C48H76O19_Hexopyranose, 1-O-[(3beta,5xi,9xi,18xi)-3-[(3-O-hexopyranosylhexopyranuronosyl)oxy]-28-oxoolean-12-en-28-yl]-	Triterpene saponins
56	13.61	291.0954163	C_11_H_17_NO_8_	N-fructosyl pyroglutamate	N-fructosyl amino acids
57	13.61	610.1533845	C_27_H_30_O_16_	Rutoside Syn. Rutin	Flavonoid-O-glycosides
58	13.61	284.075684	C_10_H_12_N_4_O_6_	Xanthosine	Purine nucleosides
	13.62	284.075684	C_10_H_12_N_4_O_6_		
59	13.62	152.0334253	C_5_H_4_N_4_O_2_	Xanthine	Xanthines
60	13.73	152.0684734	C_5_H_12_O_5_	L-arabitol (Isomer II)	Sugar alcohols
61	13.79	454.3446952	C_30_H_46_O_3_	NCGC00380944-01_C30H46O3_(3beta,5xi,9xi,13alpha,17alpha,18xi)-3-Hydroxy-13,28-epoxyurs-11-en-28-one Syn. 3-Hydroxy-11-Ursen-28,13-Olide	Triterpenoids
62	13.97	456.3603452	C_30_H_48_O_3_	Ursolic acid (Isomer I)	Triterpenoids
63	14.10	770.2269433	C_34_H_42_O_20_	7-Methylquercetin-3-galactoside-6″-rhamnoside-3‴-rhamnoside	Flavonoid -O-glycosides
64	14.21	221.0899371	C_8_H_15_NO_6_	N-acetylmannosamine	N-acyl-alpha-hexosamines
65	14.29	180.063388	C_6_H_12_O_6_	Psicose	Monosaccharides
66	14.69	456.3603452	C_30_H_48_O_3_	Ursolic acid (Isomer II)	Triterpenoids
67	14.76	165.0789785	C_9_H_11_NO_2_	Phenylalanine	Amino acids
68	14.90	204.0898776	C_11_H_12_N_2_O_2_	Tryptophan	Indolyl carboxylic acids and derivatives
69	14.93	283.0916684	C_10_H_13_N_5_O_5_	Guanosine	Purine nucleosides
70	14.93	742.1956431	C_32_H_38_O_20_	NCGC00180410-02!3-[(2S,3R,4S,5S,6R)-6-[[(2R,3R,4R,5R,6S)-3-[(2S,3R,4R)-3,4-dihydroxy-4-(hydroxymethyl)oxolan-2-yl]oxy-4,5-dihydroxy-6-methyloxan-2-yl]oxymethyl]-3,4,5-trihydroxyoxan-2-yl]oxy-2-(3,4-dihydroxyphenyl)-5,7-dihydroxychromen-4-oneSyn. Quercetin 3-(2R-apiosylrutinoside)	Flavonoid-O-glycosides
71	15.04	131.0946286	C_6_H_13_NO_2_	Isoleucine	Isoleucine and derivatives
72	15.11	104.107539	C_5_H_14_NO	Choline (Isomer I)	Cholines
73	15.14	756.2112932	C_33_H_40_O_20_	2-(3,4-Dihydroxyphenyl)-3-[(2S,3R,4S,5S,6R)-4,5-dihydroxy-3-[(2R,3R,4R,5R,6S)-3,4,5-trihydroxy-6-methyloxan-2-yl]oxy-6-[[(2R,3R,4R,5R,6S)-3,4,5-trihydroxy-6-methyloxan-2-yl]oxymethyl]oxan-2-yl]oxy-5,7-dihydroxychromen-4-one Syn. Quercetin 3-O-rutinoside-(1-2)-O-rhamnoside	Flavonoid-O-glycosides
74	15.19	182.079038	C_6_H_14_O_6_	D-sorbitol	Sugar alcohols
75	15.27	117.0789785	C_5_H_11_NO_2_	Betaine	Alpha amino acids
76	15.39	135.0544952	C_5_H_5_N_5_	Adenine	6-Aminopurines
77	15.59	104.107539	C_5_H_14_NO	Choline (Isomer II)	Cholines
78	15.64	180.063388	C_6_H_12_O_6_	Mannose	Hexoses
79	15.78	212.0896027	C_7_H_16_O_7_	Perseitol	Sugar alcohols
80	15.79	137.0476784	C_7_H_7_NO_2_	Trigonelline	Alkaloids and derivatives
81	15.84	145.0851265	C_5_H_11_N_3_O_2_	4-Guanidinobutanoic acid	Gamma amino acids and derivatives
82	16.49	293.1474519	C_12_H_23_NO_7_	N-fructosyl isoleucine	N-fructosyl amino acids
	16.52	293.1474519	C_12_H_23_NO_7_		
83	17.24	342.1162113	C_12_H_22_O_11_	Trehalose	Disaccharides
84	17.25	342.1162113	C_12_H_22_O_11_	Maltose	Oligosaccharides
85	17.25	342.1162113	C_12_H_22_O_11_	Isomaltulose	Oligosaccharides
86	17.25	342.1162113	C_12_H_22_O_11_	Melibiose	Oligosaccharides
87	17.29	147.0531577	C_5_H_9_NO_4_	L-glutamic acid	Glutamic acid and derivatives
88	18.13	146.0691421	C_5_H_10_N_2_O_3_	Glutamine	D-alpha-amino acids
89	18.30	344.1318613	C_12_H_24_O_11_	Maltitol Syn. 4-O-alpha-D-Glucopyranosyl-D-glucitol	Hexoses
90	19.31	504.1690346	C_18_H_32_O_16_	Maltotriose	Oligosaccharides
91	19.33	504.1690346	C_18_H_32_O_16_	Raffinose	Oligosaccharides
92	20.64	179.0793724	C_6_H_13_NO_5_	D-mannosamine	Hexoses
93	20.79	666.2218579	C_24_H_42_O_21_	Tetrasaccharides (Hex-Hex-Hex-Hex)	Oligosaccharides

## Data Availability

The data that support the findings of this study are available from the corresponding author upon reasonable request.

## Supplementary Materials

The following are available online at https://www.mdpi.com/article/10.3390/plants10112417/s1, Table S1: List of compounds putatively identified by LC-HRMS/MS in the extract of *Chenopodium quinoa* sprouts. Table S2: List of compounds putatively identified by LC-HRMS/MS in the extract of *Chenopodium quinoa* seeds.
